# Short-Term and Long-Term Survival and Virulence of *Legionella pneumophila* in the Defined Freshwater Medium Fraquil

**DOI:** 10.1371/journal.pone.0139277

**Published:** 2015-09-25

**Authors:** Nilmini Mendis, Peter McBride, Sébastien P. Faucher

**Affiliations:** Department of Natural Resource Sciences, Faculty of Agricultural and Environmental Sciences, McGill University, Sainte-Anne-de-Bellevue, QC, Canada; Purdue University, UNITED STATES

## Abstract

*Legionella pneumophila* (*Lp*) is the etiological agent responsible for Legionnaires’ disease, a potentially fatal pulmonary infection. *Lp* lives and multiplies inside protozoa in a variety of natural and man-made water systems prior to human infection. Fraquil, a defined freshwater medium, was used as a highly reproducible medium to study the behaviour of *Lp* in water. Adopting a reductionist approach, Fraquil was used to study the impact of temperature, pH and trace metal levels on the survival and subsequent intracellular multiplication of *Lp* in *Acanthamoeba castellanii*, a freshwater protozoan and a natural host of *Legionella*. We show that temperature has a significant impact on the short- and long-term survival of *Lp*, but that the bacterium retains intracellular multiplication potential for over six months in Fraquil. Moreover, incubation in Fraquil at pH 4.0 resulted in a rapid decline in colony forming units, but was not detrimental to intracellular multiplication. In contrast, variations in trace metal concentrations had no impact on either survival or intracellular multiplication in amoeba. Our data show that *Lp* is a resilient bacterium in the water environment, remaining infectious to host cells after six months under the nutrient-deprived conditions of Fraquil.

## Introduction

Identified as the etiological agent of Legionnaires’ disease in the late 1970’s, *Legionella pneumophila* (*Lp*) is a Gram-negative, water-borne bacterium [1]. Inhalation of *Legionella*-contaminated aerosols can lead to Legionellosis, which is comprised of the mild, flu-like Pontiac fever and the more serious pneumonia, Legionnaires’ disease [2, 3]. *Legionella* species are often found in freshwater bodies as well as an assortment of man-made water distribution systems [4–7] with the exception of *Legionella longbeachae* whose presence in potting soils is linked to the incidence of Legionnaires’ disease in Australia and New Zealand [8]. In Europe and North America, *Lp* is responsible for over 90% of reported Legionellosis cases [9]. Public health concerns related to *Lp* are mainly associated with its contamination of cooling towers and other man-made water distribution systems [10].

In the natural or man-made water environment, *Lp* can be found in a motile planktonic state, a sessile state within mixed species biofilms, growing intracellularly in amoeba and in a persistent state (viable but non-culturable, VBNC) [11]. In the water niche, *Lp* is exposed to a changing environment, such as variations in temperature, concentration of dissolved oxygen, minerals, anthropogenic chemicals and organic matter [11]. The most important parameter associated with the presence of *Lp* in a given water system is the heterotrophic plate count (HPC), which measures the general contamination of a system. The higher the HPC of a system, the higher the odds are of finding *Lp* within it [12–15]. Elevated total organic content (TOC) in water distribution systems has also been correlated with an increase in the incidence of *Legionella* [16]. Presumably, amoeba are attracted to contaminated sites in water systems to feed on susceptible bacteria and multiply, providing a source of prey for *Lp* [11]. In addition, *Lp* can persist in mixed species biofilms in natural and human-made water systems [17]. Nevertheless, growth of *Lp* associated with such biofilms requires the presence of amoeba, such as *Hartmannella vermiformis* [18]. Symbiotic and competitive interactions with other bacterial species seem to have a major impact on the net number of *Lp* in the water environment [19]. As an example, the persistence of *Lp* in water is impaired by the presence of *Pseudomonas aeruginosa* [20, 21]. Physico-chemical factors, such as temperature, pH, and the concentration of dissolved metals also seem to have an impact on the presence of *Lp* in water systems [10, 13, 14, 22]; however, there have been conflicting results regarding the extent of their effects on *Lp* persistence in water [23–27].

To date, no standardized system has been presented to study the effect of individual environmental factors on the survival of *Lp*. Knowledge pertaining to environmental conditions that affect the presence of *Lp* in such systems has been gained mainly through prospective studies in the field [12–15, 26, 28] or by using tap water models [13, 14, 29, 30]. The impact of different environmental factors such as water temperature, pH, and the presence of trace metals has been recorded mainly as a result of on-site and environmental samplings, or using sterilized tap or distilled water [21, 26, 31–34]. As a result, the interpretation of this data does not take into account variations in water composition depending on the locality or the seasonality. Thus, there is lack of a standardized artificial freshwater medium to study *Legionella* and environmental factors affecting survival, and their subsequent effect on the virulence potential of the bacterium. A defined water medium will allow elimination of variations in water composition that are dependent on seasonality and geography. It will also provide a foundation to build water microcosm, whose complexity can be increased gradually, thus allowing the evaluation of each additional parameter separately.

We have previously shown that the sigma factor RpoS, and the stringent response is required for *Lp* to survive in water [35]. Consequently, the RpoS mutant was unable to survive in tap water, and in two defined freshwater media, DFM and Fraquil. DFM composition was derived from the salt and buffer content of the chemically defined media used for *Legionella* growth [36], and contains 50mg/L NaCl, 20 mg/L KH_2_PO_4_ and 50 mg/L KCl. Fraquil is an approximation of freshwater found in North America [37] (see Table 1 for the composition of Fraquil). Our previous results show that *Lp* survive in Fraquil as well as in tap water for 30 days, but show a slight defect in DFM [35]. Therefore, we decided to further investigate the behaviour of *Lp* in Fraquil. Here, we use Fraquil to study the impact of individual environmental factors on *Lp* in a controlled water environment. To further this goal, the consequences of changing temperature, acidity and trace metal content over a short time period were investigated by tracking the capacity of *Lp* to survive in Fraquil under these conditions, and its subsequent capacity for intracellular multiplication (ICM) in amoeba. Furthermore, we investigated the long-term influence of temperature on the survival of *Lp* and on its ICM potential.

**Table 1:** Composition of Fraquil.

Components	Concentration
CaCl_2_∙2H_2_O	0.25uM
MgSO_4_∙7H_2_O	0.15uM
NaHCO_3_	0.15uM
K_2_HPO_4_	10nM
NaNO_3_	0.1uM
FeCl_3_∙6H_2_O	10nM
CuSO_4_∙5 H_2_O	1nM
(NH_4_)_6_Mo_7_O_24_∙4 H_2_O	0.22nM
CoCl_2_∙6 H_2_O	2.5nM
MnCl_2_∙4 H_2_O	23nM
ZnSO_4_∙7 H_2_O	4nM

## Methods

### Bacterial Strains and Media

All experiments were conducted using JR32 or its derivatives. JR32 is a salt-sensitive, streptomycin-resistant, restriction negative mutant of *Legionella pneumophila* (*Lp*) strain Philadelphia 1 [38]. The *dotA* ^-^ strain, used as a negative control in intracellular multiplication (ICM) assays, is a transposon mutant carrying a mutation in the type IVb secretion system that is essential for intracellular multiplication in *Lp* [38]. Strains stored at -80°C in 10% glycerol were grown on BCYE (ACES-buffered charcoal yeast extract) agar supplemented with 0.25mg/ml L-cysteine and 0.4mg/ml ferric pyrophosphate. AYE broth (BCYE without agar and charcoal) was used as the liquid medium. The defined water medium used for water exposure experiments, Fraquil was prepared as described by Morel et al. [37] with a final iron concentration of 10nM and was filter-sterilized using a 0.2um filter (Sarstedt). Ultrapure Type 1 water (18.2 MΩ·cm at 25°C), produced with a Synergy Ultrapure Water System (EMD Millipore), was used to prepare Fraquil. The complete composition of Fraquil is presented in Table 1.

### Water Exposure Experiments

JR32 cultured on BCYE agar at 37°C for 3 days was washed three times with Fraquil and suspended in fresh Fraquil at an OD_600nm_ of 0.1. Then, 1ml of the bacterial suspension was mixed with 4ml of fresh Fraquil in a 25cm^2^ cell culture flasks. Three biological replicates were used for each experiment. To test the impact of temperature, the flasks were incubated at six temperatures: 4°C, 10°C, 25°C, 30°C, 37°C, 42°C. For water experiments with varying pH, aliquots of Fraquil were adjusted to pH 4, 5, or 6 with 0.01M HCl and *Lp* was suspended at each pH as described above. To test the effect of varying trace metal concentrations, Fraquil was prepared without the addition of trace metal (0X), by adding twice the volume of trace metals than standard Fraquil (2X) or by adding 10 times the volume of trace metals (10X). Standard Fraquil (1X metals, please refer to Table 1 for composition) was used as a control. *Lp* was suspended in each trace metal concentration as described above. For each environmental parameter tested, CFU counts on BCYE agar were used at defined time points to track survival over time.

### Intracellular Multiplication Assays

Intracellular multiplication was measured in the amoeba *Acanthamoeba castellanii* and THP-1-derived human macrophages with a multiplicity of infection (MOI) of 0.1. *A*. *castellanii* was cultured in peptone yeast glucose (PYG) broth [39]. PYG contained 20g/L proteose peptone, 1g/L yeast extract, 0.1M glucose, 0.4mM MgSO_4_, 0.05mM CaCl_2_, 0.1mM sodium citrate, 0.005mM Fe(NH_4_)_2_(SO_4_)_2_, 0.25mM Na_2_HPO_4_ and 0.25mM KH_2_PO_4_, and the pH was adjusted to 6.5 using 1M HCl. Each infection well in a 24-well plate was seeded with 5x10^5^ *A*. *castellanii* cells in 1ml of PYG. One hour prior to infecting *A*. *castellanii* with *Lp*, the media in each well was replaced with 1ml of Ac buffer (PYG without proteose peptone, yeast extract and glucose). The plate was incubated for an additional hour at 30°C before introducing approximately 5 x 10^4^ *Lp* to each well. THP-1 monocytes were cultured in RPMI (GIBCO) supplemented with L-glutamine and 5% FBS. For the THP-1 infection, 5x10^5^ cells treated with 10^−7^ M phorbol 12-myristate 13-acetate (PMA) were seeded into a 24-well plaque in 1ml of RPMI 3 days prior to infection and left to incubate at 37°C in 5% CO_2_. One hour prior to infecting the macrophages with *L*. *pneumophila*, the media in each well was replaced with fresh RPMI. THP-1 cells were infected with approximately 5 x 10^4^ *Lp* to each well.

The infection wells were sampled daily to detect the extracellular increase of CFUs relative to time zero. The laboratory wild type JR32 was used as a positive control, while the *dotA* ^-^ mutant, deficient in intracellular replication, served as a negative control. Both control strains were grown on BCYE agar and suspended in AYE broth at an OD_600_ of 0.1, and were then further diluted 10 fold to obtain an approximate OD_600_ of 0.01. 2μl of this final solution was used to infect the cells.

For bacterial samples originating from water experiments testing temperature, pH or trace metal content, a CFU count was done 3 days prior to the infection. On the day of the infection assay, this count was used to determine the volume representing 1 x 10^4^ bacterial cells, thus resulting in an MOI of 0.1. When necessary, bacteria were diluted in Fraquil.

### Statistical analysis

The graphs show the average of at least three biological replicates and the standard deviation. We used unpaired the one tail Student’s T-test to access statistical significance.

## Results

### Short-term effect of temperature on the survival of *Lp* and subsequent ICM potential

**Fig 1:**
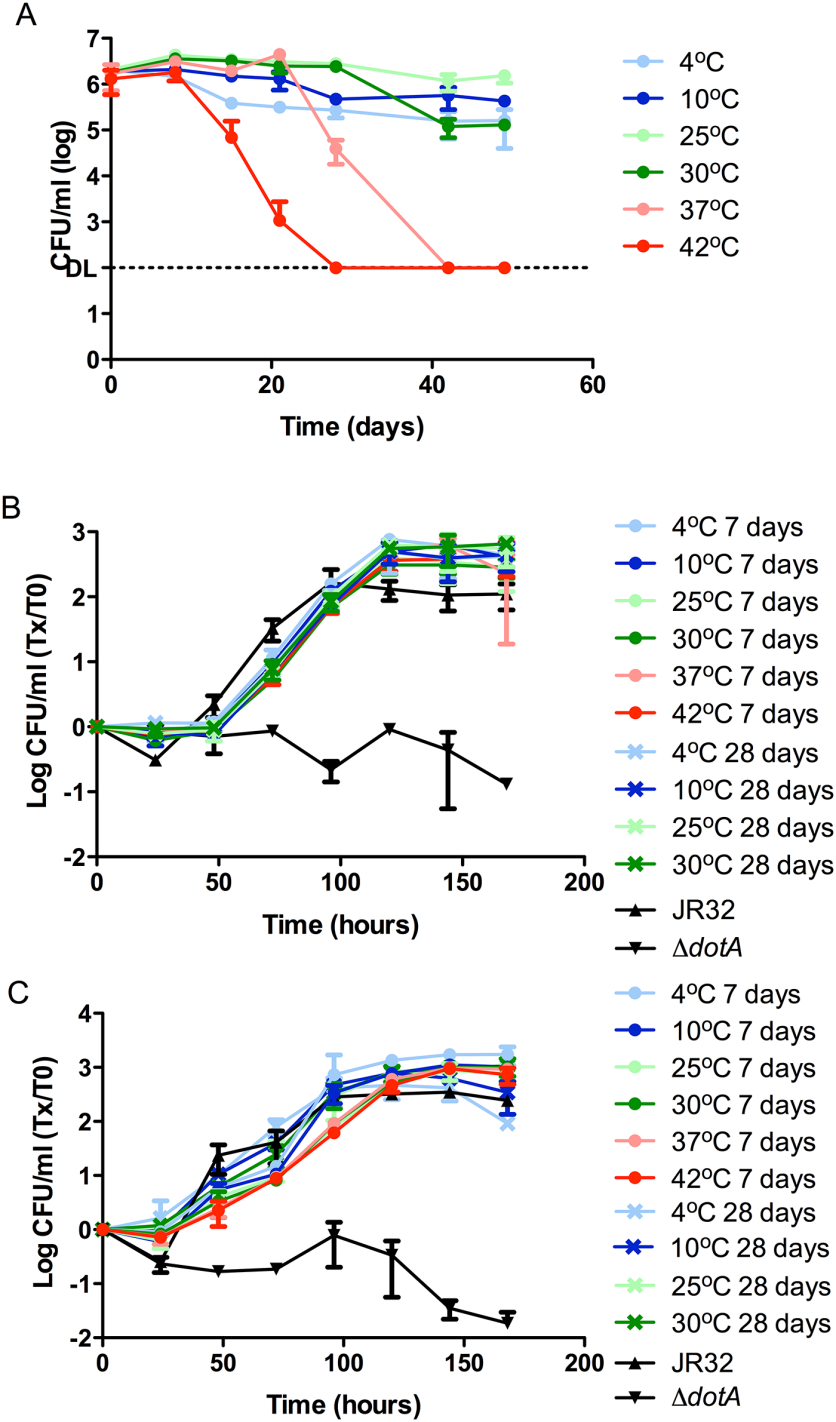
Impact of temperature on the short-term survival of *Lp* in Fraquil and intracellular multiplication (ICM) after exposure to different temperatures. **A)** The JR32 strain was exposed to Fraquil at six different temperatures. Weekly CFU counts were performed to track survival. DL, detection limit. At 4°C, 10°C, 37°C and 42°C, CFU counts from 8 days were statistically different (*P*≤0.05) than CFU counts at 25°C. At 30°C, CFU counts from day 42 were statistically different that CFU counts at 25°C. **B)** *A*. *castellanii* was infected with JR32 that had been exposed to the respective temperatures tested for 7 days or 28 days at an MOI of 0.1. Daily CFU counts monitored the ICM inside amoeba and are presented as the ratio over CFU counts at day 0. JR32 from BCYE was used as the positive control and *dotA* ^-^ was used as the negative control. **C)** Cultured human macrophages (THP-1) were infected with JR32 that had been exposed to the respective temperatures tested for 7 days or 28 days at an MOI of 0.1. Daily CFU counts monitored the ICM and are presented as the ratio over CFU counts at day 0. JR32 from BCYE was used as the positive control and *dotA* ^-^ was used as the negative control.

We tested the survival of *Lp* in Fraquil, hereafter called water, exposed to six different temperatures ranging from refrigeration to the high end of the temperature spectrum recorded as supporting *Legionella* growth [40]: 4°C, 10°C, 25°C, 30°C, 37°C and 42°C. Using CFU counts, the survival of the JR32 strain was monitored for 49 days (Fig 1A). It was clear that changes in temperature had a definite impact on the survivability of *Lp* in this water system. The 42°C experimental condition resulted in the fastest CFU decline starting at 15 days, reaching the detection limit of 100 CFU/ml in all three replicates after 28 days. At 37°C, which is in the range of optimal growth temperatures for *Legionella*, bacterial numbers were stable for approximately 1 month after which the CFU counts decreased dramatically reaching the detection limit at the end of 42 days. A moderate dip in the CFU counts was observed at 30°C at day 42, while *Lp* incubated at 25°C showed no decrease in bacterial counts during the 49-day tracking period testing survivability in water. At 10°C, *Lp* seems to maintain relatively stable bacterial counts. The set of samples at 4°C showed a small decrease in CFU counts after approximately two weeks and then stabilized for the remainder of the time tested (49 days). Our results show that even moderate temperatures between 30°C to 42°C significantly impact the survivability of *Lp* in a minimal water system (Fig 1A).

Following water exposure, we tested the infectivity of *Lp* incubated at different temperatures towards *Acanthamoeba castellanii*, a natural host of *Legionella* found in natural and man-made water systems [41]. The *dotA* ^-^ mutant strain was used as a negative control in the infection assays due to its deficiency in intracellular multiplication (ICM) in *A*. *castellanii* as a result of a Type IVb secretion system defect [38]. To detect any changes of virulence potential in response to different incubation times in water, we used bacteria that had been exposed to the respective temperatures for both 7 and 28 days (Fig 1B). *Lp* exposed to 37°C and 42°C was not used in the 28 day infections since the CFU counts were either too low (37°C) or non-existent (42°C). The volume of the 37°C samples required to infect the cells at an MOI of 0.1 was too large and would have compromised the dynamics of the infection assay, thus making the results unreliable. At the end of the infection cycle (168 hours), there was no significant difference in the CFU increase during infection between the different temperatures. Moreover, no significant differences were observed between 7-day and 28-day exposure times. The ICM potential in cultured human macrophages was also tested in parallel (Fig 1C). As for the infection of amoeba, there were no significant differences between the positive control and the bacterial cultures originating from water. Since no significant difference was observed between temperature-treated samples in macrophages, subsequent ICM experiments were conducted exclusively in *A*. *castellanii*, which is more relevant in the context of the water environment.

These infection assay results show that *Lp* is able to maintain its ICM potential for at least 28 days post-incubation in the nutrient-deficient water environment at temperatures ranging from 4°C to 30°C, and retains ICM at least 7 days after exposure to water at 37°C and 42°C, temperatures that are environmentally relevant (Fig 1B and 1C).

### Effect of trace metal concentrations on the survival and subsequent ICM of *Lp*

**Fig 2:**
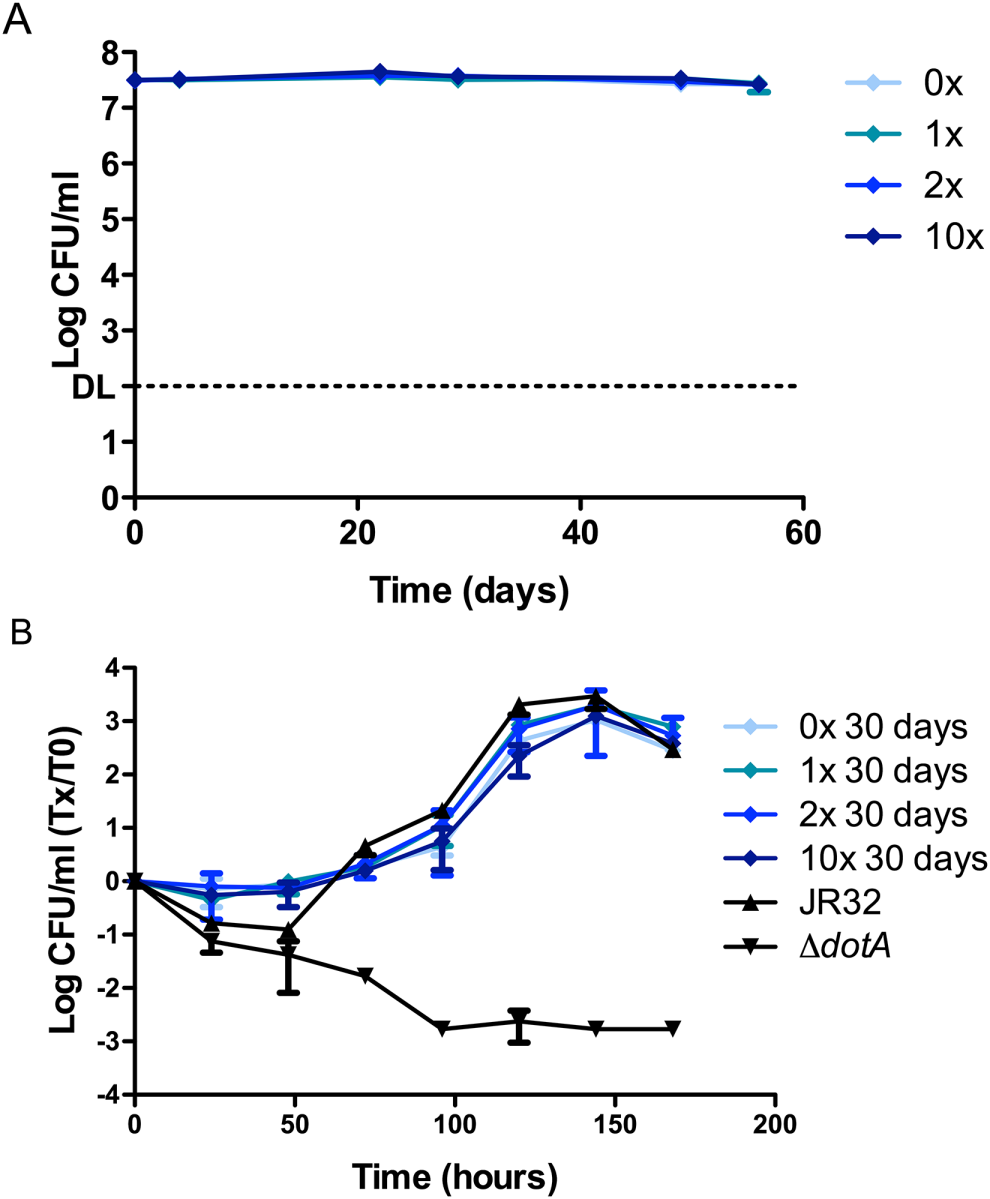
Survival of *Lp* at different trace metal concentrations and subsequent intracellular multiplication (ICM). **A)** The JR32 strain of *Lp* was exposed to Fraquil at four different metal concentrations: no addition of trace metals (0X), standard Fraquil (1X), double the quantity of trace metals (2X) and 10 times the quantity of trace metals (10X) than in standard Fraquil. Weekly CFU counts were performed to track survival. DL, detection limit. **B)** *A*. *castellanii* was infected with JR32 that had been exposed to the respective levels of trace metals tested for 30 days at an MOI of 0.1. Daily CFU counts monitored the ICM inside amoeba and are presented as the ratio over CFU counts at day 0. JR32 from BCYE was used as the positive control and *dotA* ^-^ was used as the negative control.

To test whether a water environment with a reduced level of trace metals impairs the ability of *Lp* to survive, or if an increase in these metal concentrations may help it survive better, we tested four different trace metal concentrations in Fraquil; namely without the addition of trace metals (0X), standard Fraquil (1X), double the concentration of trace metals (2X) and 10 times the concentration of trace metals (10X) used in standard Fraquil (Fig 2A). Over a period of 56 days, there were no observable significant differences in the CFU counts between *Lp* exposed to different trace metal concentrations.

The ICM capacity of *Lp* exposed for 30 days to different trace metal concentrations in Fraquil was tested (Fig 2B). The ICM rate of the water exposed *Lp* samples were comparable to that of *Lp* cultured on BCYE agar (positive control). Furthermore, no difference was observed in ICM between *Lp* exposed to different trace metal concentrations. Therefore, it would seem that exposure to water containing a higher amount of trace metals, or a lack thereof, does not impact the ICM of *Lp* in the amoebal host.

### Effect of pH on the survival and subsequent ICM of *Lp*

**Fig 3:**
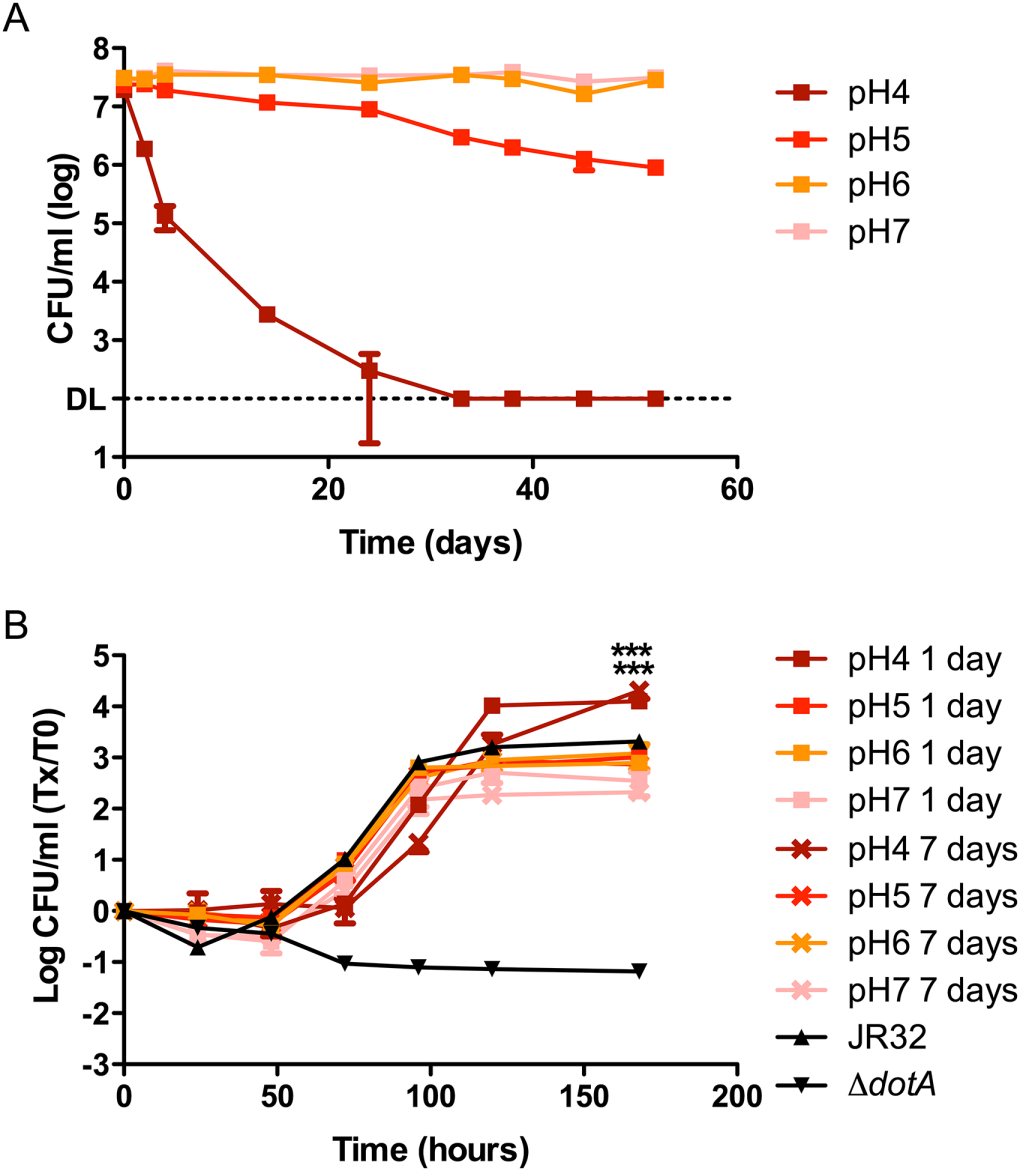
Impact of pH on the short-term survival of *Lp* in Fraquil and intracellular multiplication (ICM) after exposure to different pH. **A)** The JR32 strain of *Lp* was exposed to Fraquil at four different pH. Weekly CFU counts were performed to track survival. DL, detection limit. At pH 4 and pH 5, CFU counts from 2 days are statistically different (*P*≤0.05) than CFU counts at pH 7. At pH 6, CFU counts from 38 days are statistically different than CFU counts at pH 7. **B)** *A*. *castellanii* was infected with JR32 that had been exposed to the respective pH tested for either 1 day or 7 days at an MOI of 0.1. Daily CFU counts monitored the ICM inside amoeba and are presented as the ratio over CFU counts at day 0. JR32 from BCYE was used as the positive control and *dotA* ^-^ was used as the negative control. *** *P*≤ 0.005 versus control.

The survival of *Lp* in Fraquil at three different pH levels (4, 5 & 6) was tested in addition to the standard Fraquil whose pH hovers around 7.3. The pH experiment was conducted at 25°C since it is a permissive temperature for the survival of *Lp* in water (Fig 1A). Changing the pH had a much more drastic effect on the survivability of *Lp* compared to temperature variations. When the water medium was adjusted to a pH value of 4, CFU counts steadily declined to the detection limit within 33 days (Fig 3A). At a pH value of 5, the CFU counts started to visibly decrease after 24 days of incubation in the defined water medium. The higher pH values of 6 and 7.3 of normal Fraquil resulted in no loss of culturability over the course of the experiment (52 days).

The virulence potential of *Lp* incubated in acidified Fraquil was tested by following the intracellular multiplication (ICM) in the same manner used for the temperature and trace metal variation experiments in *A*. *castellanii*. Since the CFU counts decreased dramatically in the first week of exposure to pH 4, we used *Lp* that had been exposed to water for 24 hours and for 7 days to test the ICM in amoeba. Interestingly, compared to samples at pH 7 and the positive control, incubation in Fraquil at pH 4 at both exposure times resulted in a statistically significant, 1 log increase of CFU counts at the end of a 168 hour infection cycle (Fig 3B). The ICM of *Lp* exposed to pH 5, pH 6 and standard Fraquil produced a similar increase in bacterial load upon infection of *A*. *castellanii* compared to *Lp* originating from rich media.

### Long-term survival of *Lp* in water and subsequent effect on ICM

**Fig 4:**
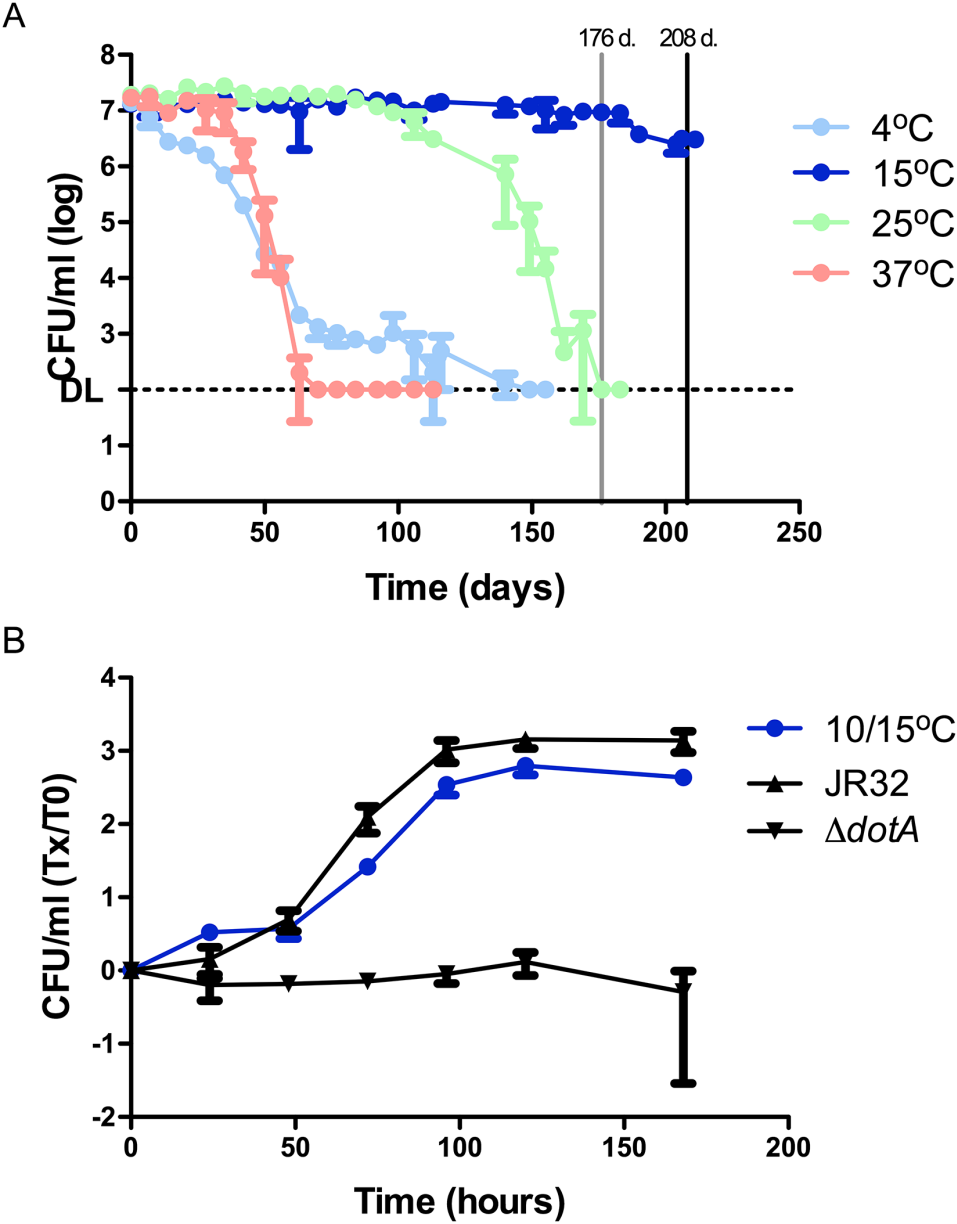
Long-term survival of *Lp* in Fraquil and intracellular multiplication (ICM) after long-term exposure to a moderate temperature. **A)** The JR32 strain was exposed to Fraquil and CFU counts were used to track survival over a 211-day time period. Weekly CFU counts were performed to track survival. DL, detection limit. The vertical lines indicate the time points at which the temperature of incubator was reduced from 15°C to 10°C (grey– 176 days) and when samples were harvested to infect amoeba (black– 208 days). At 4°C, CFU counts from 14 days are statistically different (*P*≤0.05) than CFU counts at 15°C. At 25°C, CFU counts from 113 days are statistically different than CFU counts at 15°C. At 37°C, CFU counts from 42 days are statistically different than CFU counts at 15°C. **B)** *A*. *castellanii* was infected with JR32 that had been exposed to 15°C for 176 days and 10°C for an additional 32 days at an MOI of 0.1. Daily CFU counts monitored the ICM inside amoeba and are presented as the ratio over CFU counts at day 0. JR32 from BCYE was used as the positive control and *dotA* ^-^ was used as the negative control.

To test whether *Lp* was able to survive in Fraquil over a longer period of time, inoculums were tested at four temperatures (4°C, 15°C, 25°C and 37°C). The lower end of the temperature spectrum was used, since higher temperatures were found to be detrimental to *Lp* in the short-term (Fig 1A). We used 37°C as a short-term survival temperature control. CFU counts were tracked over a period of 211 days in total (Fig 4A). As was expected (Fig 1A), the fastest decline in the bacterial population was observed at 37°C, where no CFUs could be detected on agar plates after 70 days. At 25°C, inoculums were stable for 98 days after which the CFU counts started to decrease, reaching the detection limit by 176 days. At 4°C, the CFU counts reached the detection limit by 149 days. We tested long-term survival at 15°C instead of 10°C. *Lp* was initially exposed to 15°C for 176 days, but the flasks were incubated at 10°C for the following 32 days prior to testing ICM. On the long term, *Lp* survived at 15°C even better than at 25°C showing no significant decrease in CFU counts until 183 days.

To test whether the ICM capacity of *Lp* had been affected negatively or positively after a long incubation period in Fraquil, *A*. *castellanii* was infected with bacteria that had been incubated in water for a total of 208 days, 176 days at 15°C and another 32 days at 10°C. While the increase in bacterial counts during the infection is statistically lower than that of *Lp* originating from rich media, *Lp* seems to retain most of its capacity for ICM even after an extended time in water and in the absence of any additional nutrients (Fig 4B).

## Discussion

A variety of water distribution systems can harbour *Lp* including shower heads, hot water tubs and hot water tanks [22, 42, 43]. In fact, the design of some systems can further favour *Legionella* contamination and persistence in water, either by cooler bodies of water within hot water systems or dead legs creating areas of stagnant water [25, 44]. For example, domestic water heating units in Quebec City, Canada, that are powered by electricity were shown to contain an area of water that was at a significantly lower temperature than the rest of the tank, thus, allowing the survival and growth of *Legionella* species in contaminated units [22, 25].

In this study, we have used Fraquil, to explore the effect of temperature, pH and trace metal concentration on the survival of *Lp* and on its subsequent capacity to grown inside amoeba. It is noteworthy that the JR32 laboratory strain was used for this study and that environmental isolates of *Lp* may behave differently in Fraquil. We are currently screening multiple clinical and environmental isolates.

Several studies report finding the bacterium at temperatures exceeding 50°C in the environment while others have shown that *Lp* is metabolically active above 45°C [26, 27, 40, 45–47]. In rich media, *Lp* grows optimally under laboratory conditions between 25°C and 37°C. Early studies also showed that *Lp* is able to multiply in unsterilized tap water containing amoeba between 25°C and 42°C over a period of 21 days, but that it could not replicate in the absence of amoeba at temperatures above 37°C [29, 31, 32]. Wadowsky et al. [29] demonstrated that an environmental isolate survived for 28 days in distilled water at 35°C. An early study by Dennis et al. [48] showed that *Lp* is more temperature tolerant on the short term than *Pseudomonas* and *Micrococcus* species, both found with *Lp* in water distribution systems. This characteristic has been taken advantage of when isolating *Legionella* spp. from environmental sources, using a mild heat treatment to increase isolation [49, 50]. Therefore, we expected to find that *Lp* would be relatively resistant to heat in Fraquil, the artificial freshwater medium used in this study. To our surprise, we found that *Lp* survive for only 6 and 3 weeks at 37°C and 42°C, respectively. Our study suggests that the reported persistence of *Lp* in water systems at high temperatures is positively affected by variables other than temperature; these variables may include protection inside thermophilic amoebal hosts, amoeba cysts or inside biofilm [31, 51, 52].

The steady decrease in CFUs observed at 4°C (Fig 4A) is consistent with recorded loss of culturability at 4°C, but the survival or culturablility periods vary according to different groups [28]. For example, Wadowsky et al. [29] observed that storing environmental samples at 5°C resulted in a decrease of CFUs. In addition, the earliest reports of viable-but-nonculturable (VBNC) *Lp* cells elude to a 4°C incubation temperature inducing this state [53]. We, therefore, suspect that *Lp* enters a VBNC state in water at 4°C; however, this will require further experimentation.

A moderate water temperature of 25°C allowed *Lp* to survive approximately three months in Fraquil (Fig 4A). At 15°C, *Lp* survive for at least 208 days. Similarly, Paszko-Kolva et al. [30] show that in drinking water samples, a clinical *Lp* strain has no significant difference in the CFU counts after incubation at 15°C for approximately 200 days, but that CFU counts decreased more significantly when using creek or estuarian waters. This ability to survive over a period of several months would allow *Lp* to persist in a water system and replicate in amoebal hosts when the latter arrive into the system. Most community outbreaks of Legionnaires’ disease occur in the late summer or early fall seasons [54, 55]. A gradual increase of *Lp* in a water system over this time frame will eventually allow the bacterium to attain a concentration sufficient for disease transmission in humans during the spring and summer, and survive over the winter at lower temperature.

We demonstrate that *Lp* incubated at a temperature ranging from 4°C to 42°C were able to replicate in *A*. *castellanii* and in cultured human macrophages after exposure to water for at least one week, while *Lp* at lower temperatures maintained intracellular multiplication (ICM) capacity for at least 28 days (Fig 1B and 1C). Temperature is known to regulate the expression of virulence factors in other pathogenic bacteria like *Shigella*, *Streptococcus* and *P*. *aeruginosa* [56–58]; however, the interplay between *Lp* virulence and temperature is yet to be clearly defined. A growth temperature of 37°C was shown to increase virulence of *Lp* while growth at 24°C results in an avirulent strain in a guinea pig model establishing a first link between temperature and virulence of *Lp* [23]. More importantly, the avirulence observed at 24°C was corrected when the temperature was shifted to 37°C [23]. In contrast, another study showed that *Lp* grown at 25°C is more lethal to guinea pig macrophages *in vitro* [24]. While contradictory, both studies show a definite effect of temperature on the virulence of *Lp*. In addition, pili and flagella are expressed in *Lp* in a temperature-dependent manner, and the expression of both structures have been linked to the bacterium’s virulence in host cells [59–63]. The type II secretion system is involved in the production of the type IV pili in *Lp* [59]. Soderberg et al. [64] reported that the type II secretion system of *Lp* allowed survival in water at 4°C and 17°C and also played a role in intracellular multiplication (ICM) in amoeba at 22°C-25°C. Moreover, pili were shown to play a role in adherence to both amoeba and human cells [61]. Flagella expression has also been demonstrated to be temperature regulated and is implicated in virulence [60, 62].

Moreover, we demonstrate that *Lp* incubated at 15°C were able to infect and kill amoeba after resting in Fraquil for approximately six months, albeit at a slightly lower rate than the positive control (Fig 4B). Our results suggest that, even in the absence of its natural hosts and lack of sufficient nutrients for growth (i.e. without the contamination of water systems by organic material), *Lp* may still pose a significant threat to public safety for a long period of time, as it remains virulent and competent for ICM. Therefore, *Lp* is able to easily linger in a clean water system for many months before coming into contact with its amoeba prey.

The presence of some metals has been linked to a decrease of *Legionella* in water systems while other metals are deemed as contributors to its survival [65]. Copper and silver ions are used to disinfect water distribution systems and have been studied for their negative effects on *Legionella* species, and they are known to decrease contamination levels in conjunction with other treatments [66, 67]. Indeed, a negative correlation is observed between the incidence of *Lp* and elevated trace levels of copper ions [13, 14]. In addition, as observed in the cases of many other bacteria, *Lp* requires iron for optimal growth on artificial media and in host cells as evidenced by its multiple iron acquisition systems [68–73]. In accordance, *Lp* contamination is positively correlated with higher concentrations of iron in water systems [12–14]. Furthermore, higher concentrations of manganese, zinc and cobalt have also been shown to correlate positively with *Lp* contamination of water systems [13, 36].

No survival defect (Fig 2A) was observed in any of the trace metal variations that were tested. While a positive relationship has been shown between the concentrations of manganese, iron and zinc, and the presence of *Lp* in hot water systems in some studies [13], no such correlation was found in others [12, 14]. There was no difference in ICM after 30 days of exposure to the respective trace metal concentrations (Fig 2B). The infection itself was performed in Ac buffer, which contains 5nM of iron. Therefore, any negative effects on ICM caused by the lack of iron in Fraquil without trace metals may have been rescued upon exposure of *Lp* to Ac buffer. The Ac buffer does not contain the other trace metals found in Fraquil; therefore, it is possible to conclude that the trace metals in Fraquil other than iron do not affect the ICM of *Lp* in the concentrations tested. Our results suggest that the impact of the concentration of metals on the survival and growth of *Lp* in water systems is linked to other conditions, potentially the presence of susceptible amoeba hosts.

Once inside a host cell, *Lp* grows within the *L* *egionella* containing vacuole (LCV) which evades the normal endocytic pathway by hijacking the host cell machinery [74]. While virulent *Lp* disturbs the acidification of the LCV, the recruitment of V-ATPases that carry out the acidification is not fully blocked in a small number of cases [75, 76]. Therefore, there is a biological need for *Lp* to have and to use mechanisms to survive pH stress. This may explain the relative hardiness of *Lp* toward varying pH that has been observed in nature and in man-made water systems. In fact, environmental sampling shows that *Lp* can be recovered at a wide range of pH (5.5 to 8.1) [26]. Wadowsky et al. [29] also found that an environmental isolate of *Lp* was able to replicate in filter-sterilized tap water from pH 5.5–9.2. Furthermore, isolating environmental strains of *Legionella* is known to be greatly enhanced by an acid treatment, suggesting that it is relatively more tolerant to acid than other bacteria found in water [77, 78]. *Lp* has been shown to tolerate a pH 2 treatment for at least 30 minutes [79]. More recently, the *Lp* genome was shown to encode carbonic anhydrases [80]. These enzymes are involved in pH regulation and may have a role in the bacterium’s survival inside the LCV, further supporting a relatively high level of tolerance to pH in *Legionella* species [80].

A low pH of 4 significantly affected the survival of *Lp* in the water environment, resulting in no CFU counts at the end of one month. It is conceivable that *Lp*, already under the stress of adapting to the nutritionally poor water environment, is unable to cope with the additional stress of an acidic pH. Variations in pH are known to cause the precipitation of some trace metals and have been used to evaluate trace metal contents in environmental samples [81, 82]. A more acute deprivation of metal cofactors caused by precipitation reactions may be responsible for the sensitivity of *Lp* at low pH, but this hypothesis is negated by Fig 3A showing no survival defect when no trace metals are added to Fraquil. Moreover, exposure to pH 4 for 24 hours or seven days resulted in higher ICM (Fig 3B). Another study demonstrated that a pH 6.5 acid treatment over a 24 hour time period provided increased resistance to a second pH stress, to oxidative stress, and induced virulence in previously non-virulent strains [83]. It is possible that prior exposure to pH 4 primes *Lp* for the intracellular environment that is reported to attain a pH value of 5.6 [75, 84]. This possibility will require further study.

## Conclusions

We have successfully used Fraquil to investigate the survival of *Lp* in water. Temperature and pH were found to have a determinant effect on the survival, but the trace metal concentration does not impact survival of *Lp*. The ICM of *Lp* seems to increase in response to low pH, but the concentration of trace metals and temperature seem to have little effect on its ICM capacity. Importantly, our results show that *Lp* retains its ability to infect host cells after long-term survival in Fraquil. Our results support the use of Fraquil as a defined freshwater medium to study the biology of *Lp* in water systems, such as the transcriptomic response of *Lp* to Fraquil [85], and to facilitate the interpretation of datasets originating from different research groups.
